# Hyperspectral optical coherence tomography for in vivo visualization of melanin in the retinal pigment epithelium

**DOI:** 10.1002/jbio.201900153

**Published:** 2019-08-13

**Authors:** Danielle J. Harper, Thomas Konegger, Marco Augustin, Kornelia Schützenberger, Pablo Eugui, Antonia Lichtenegger, Conrad W. Merkle, Christoph K. Hitzenberger, Martin Glösmann, Bernhard Baumann

**Affiliations:** ^1^ Center for Medical Physics and Biomedical Engineering Medical University of Vienna Vienna Austria; ^2^ Institute of Chemical Technologies and Analytics, TU Wien Vienna Austria; ^3^ Core Facility for Research and Technology University of Veterinary Medicine Vienna Austria

**Keywords:** hyperspectral imaging, melanin, optical coherence tomography, optical spectroscopy, retinal pigment epithelium, white light

## Abstract

Previous studies for melanin visualization in the retinal pigment epithelium (RPE) have exploited either its absorption properties (using photoacoustic tomography or photothermal optical coherence tomography [OCT]) or its depolarization properties (using polarization sensitive OCT). However, these methods are only suitable when the melanin concentration is sufficiently high. In this work, we present the concept of hyperspectral OCT for melanin visualization in the RPE when the concentration is low. Based on white light OCT, a hyperspectral stack of 27 wavelengths (440‐700 nm) was created in post‐processing for each depth‐resolved image. Owing to the size and shape of the melanin granules in the RPE, the variations in backscattering coefficient as a function of wavelength could be identified—a result which is to be expected from Mie theory. This effect was successfully identified both in eumelanin‐containing phantoms and in vivo in the low‐concentration Brown Norway rat RPE.

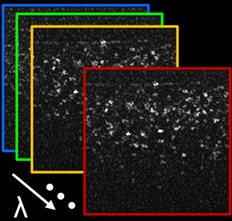

## Graphical abstract

The exact role of melanin in the retinal pigment epithelium (RPE) is still under debate. In recent years, optical techniques based on absorption and depolarization have been used for visualization of melanin in the RPE to try to solve some of these open questions. However both of these techniques rely on the melanin concentration to be sufficiently high. In this work, we have developed a hyperspectral extension to optical coherence tomography to visualize melanin in low concentrations, as is the case in the Brown Norway rat RPE.

## 1 INTRODUCTION

The protective effects of melanin on ocular cells and tissues make it an interesting heteropolymer. By a combination of physical and biochemical mechanisms, the pigment simultaneously acts as a photo‐screen and an antioxidant 1. The pigmented middle layer of the eye, known as the uvea, consists of the iris and ciliary body in the anterior eye, and the choroid in the posterior eye. In this layer, the melanin acts as an absorber for any stray light either reflected within the eye or transmitted through the sclera, improving the contrast of the final retinal image. Also located in the posterior eye is the retinal pigment epithelium (RPE). Alongside its absorption properties, the melanin in this layer is thought to play a role in many additional processes. Here, the melanin assists in the maintenance of visual function and serves as a site for sub‐cellular molecular interactions 2, 3. Owing to the close functional relationship of the RPE to the photoreceptors, it has been suggested that a change in RPE melanin content may be associated with diseases such as age‐related macular degeneration 4.

As the RPE is separated from the also melanin‐rich choroid by only Bruch's membrane (a 2‐ to 4‐μm‐thick layer in humans, 5 and even thinner in the rodent 6, 7), in vivo analysis of the melanin in the RPE alone has proven challenging. Chemical methods have been employed to study melanin in the choroid/RPE complex as a whole 8 and also in the RPE alone 9; however, such approaches are only applicable post mortem. These methods, and also those based on electron spin resonance spectroscopy 9, 10, have shown that the melanin content within RPE cells can drop down to a fifth of its initial value by the age of 80, increasing the possibility of disease 4. However, even in young subjects, the melanin concentration is not uniform across the retina 9, 11. There is therefore a need for an in vivo method for visualization of the RPE melanin, independent from that of the choroid, and optical techniques have recently been employed for this purpose 12.

Photoacoustic microscopy has been proposed as a method for visualization and/or quantification of melanin in the ex vivo human retina 13, and also in vivo in the retina of both the chick embryo 14 and the rat 15. Photothermal (PT) optical coherence tomography (OCT) has also recently demonstrated its potential as a functional technique to image melanin distribution in the RPE of the tyrosinase‐mosaic zebrafish 16. Another recent study compared the reflectivity contrast between visible light OCT and near‐infrared OCT in the RPE, indicating that the difference in melanin absorption between these two wavelength bands may lead to an intrinsic contrast, detected by the intensity ratio of the OCT images. 17. All of these techniques make use of the absorption properties of melanin as the source of image contrast and therefore the melanin concentration must be sufficiently high to achieve good image contrast. Near‐infrared autofluorescence imaging (NIR‐AF) also detects the presence of ocular melanin and can be linked to its concentration 18; however, this is only a two‐dimensional imaging modality and therefore the choroidal melanin also contributes to the observed signal. Polarization sensitive (PS) OCT, exploiting the depolarizing property of the melanin granules, has also been applied in the retina 19, 20. PS‐OCT was utilized to show a relationship between the optical pigment density in the RPE/choroid complex and the degree of polarization uniformity of the light which is backscattered from these layers 21, 22. PS‐OCT was also combined with scanning laser polarimetry 23, 24 and NIR‐AF 24, 25 for in vivo analysis of polarization properties of the RPE. However in these studies, it was not possible to directly correlate the polarization properties to the melanin concentration, most likely due to the scattering properties of the melanin granules themselves.

In order to investigate scattering properties, direct measurement of wavelength‐dependent scattering and backscattering coefficients has been demonstrated in the visible light range using low coherence spectroscopy measurements of microspheres 26. The results were in good agreement with Mie theory simulations. Since the melanin granules in the RPE are on the order of magnitude of the wavelength of visible light, it may be expected that the granules exhibit Mie regime‐like scattering behavior upon visible light illumination. In conventional OCT, only the light which is directly backscattered by the sample is detected, resulting in an imaging modality in which the intensity of the signal is dependent upon the coefficient of backscattering, *μ*_*b*_, across the entire bandwidth of the spectrum. To investigate the dependence of the backscattered signal upon wavelength, spectroscopic OCT (sOCT) is required. sOCT has been used to visualize and/or quantify chromophores such as melanin 27, bilirubin 28 and hemoglobin 29, 30, 31, mostly based on the absorption properties of the chromophores in the visible light range 32. However, sOCT has also been used to quantify the backscattering coefficient of small particles, given that the relationship of the particle size to the central wavelength is such that the modulations can be adequately sampled by the detection apparatus 33, 34, 35. The results of such studies were again consistent with Mie theory.

In this work, we propose the use of visible light sOCT to create a hyperspectral image stack to visualize melanin in the rodent RPE based on the wavelength‐dependent backscattering coefficient. To validate the results, phantoms consisting of various concentrations of melanin in silicone were created and imaged with the same system. We present both phantom and in vivo hyperspectral OCT images, and discuss the feasibility and limitations of this technique for in vivo melanin concentration measurements.

## 2 MIE THEORY

To determine the suitability of hyperspectral OCT for detection of Mie‐regime backscattering, simulations based on code by Mätzler 36 were performed. Both melanin phantoms (melanin in silicone) and the RPE (melanin in retinal tissue) were simulated, using plane wave illumination across the whole visible light range. Although the experiments use Gaussian beam illumination rather than plane wave illumination, this results only in a slight shift in spectra but the modulations remain at the same frequency 34.

Since melanin is a strong absorber in the visible light range, the wavelength‐dependent complex refractive index, **n**(*λ*), must be considered 37:

(1) $$n\left(\lambda \right)=n\left(\lambda \right)+\mathrm{i\kappa}\left(\lambda \right),$$

where *n*(*λ*) and *κ*(*λ*) are the real part and extinction coefficient of the refractive index, respectively.

The real part of the refractive index of melanin, *n*_mel_, is given as

(2) $$n_{\mathrm{mel}}\left(\lambda \right)=1.6840-1.8723\times 10^{4}\lambda^{-2}+1.0964\times 10^{10}\lambda^{-4}-8.6484\times 10^{14}\lambda^{-6},$$

across the wavelengths, *λ*, in the visible light range 38. The extinction coefficient, *κ*_mel_, was calculated from the absorption coefficient (*μ*_*a*_) of mouse RPE melanin taken from the superior retina of wild type mice 39 by

(3) $$\kappa_{\mathrm{mel}}\left(\lambda \right)=\frac{\mu_{a}\left(\lambda \right)\lambda }{4\pi },$$

40 and therefore an equation can be fitted to *κ*_mel_

(4) $$\kappa_{\mathrm{mel}}\left(\lambda \right)=9.452\times 10^{-13}\lambda^{4}-2.452\times 10^{-9}\lambda^{3}+2.447\times 10^{-6}\lambda^{2}-1.133\times 10^{-3}\lambda +0.2124,$$

across the visible light range.

The refractive index of the surrounding media was also required for the simulations. From data extracted from Figure 3 of Meichner et al. 41, the real part of the refractive index of the silicone elastomer in the visible light range, *n*_sil_, can be approximated as:

(5) $$n_{\mathrm{sil}}\left(\lambda \right)=1.527\times 10^{-7}\lambda^{2}-2.260\times 10^{-4}\lambda +1.494,$$

and since the elastomer is considered transparent at visible wavelengths, the imaginary part of the refractive index was set to zero 41, 42.

For the in vivo (RPE) case, the real part of the refractive index of the surrounding medium was considered to be an average value of 1.368 ± 0.004 43 and the imaginary part of the refractive index was also set to zero. While the non‐melanin components of the RPE do contain a small absorption coefficient in the visible light range, it is dominated by the melanin absorption 44 and therefore the imaginary component becomes negligible.

**Figure 1:**
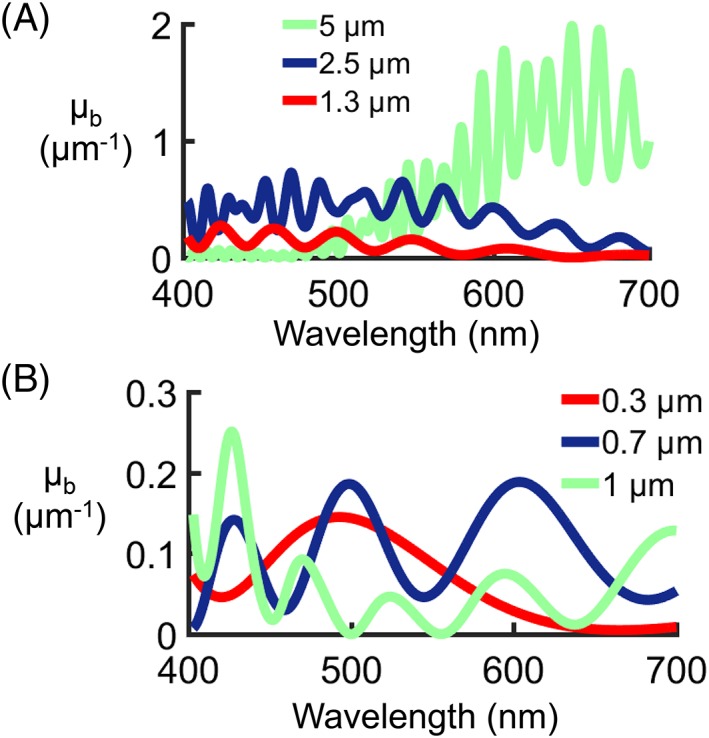
Mie theory simulations of the coefficient of backscattering, *μ*_*b*_, as a function of wavelength across the visible light range for (A) phantoms of melanin particles in silicone and (B) the rodent retinal pigment epithelium (RPE). Sizes in legend correspond to particle diameter

Using this information, the backscattering coefficient was calculated for melanin particles of varying diameters across the visible light range, examples of the results of which can be seen in Figure 1A for the melanin phantoms and Figure 1B for the rodent RPE. In both cases, modulations are observed in the visible light range. By sub‐sampling the spectra acquired by white light OCT, the presence of modulations can be detected with appropriate sampling density and axial resolution. Details of such sampling can be found in Section 3.2.

## 3 MATERIALS AND METHODS

### 3.1 Phantom preparation

**Figure 2:**
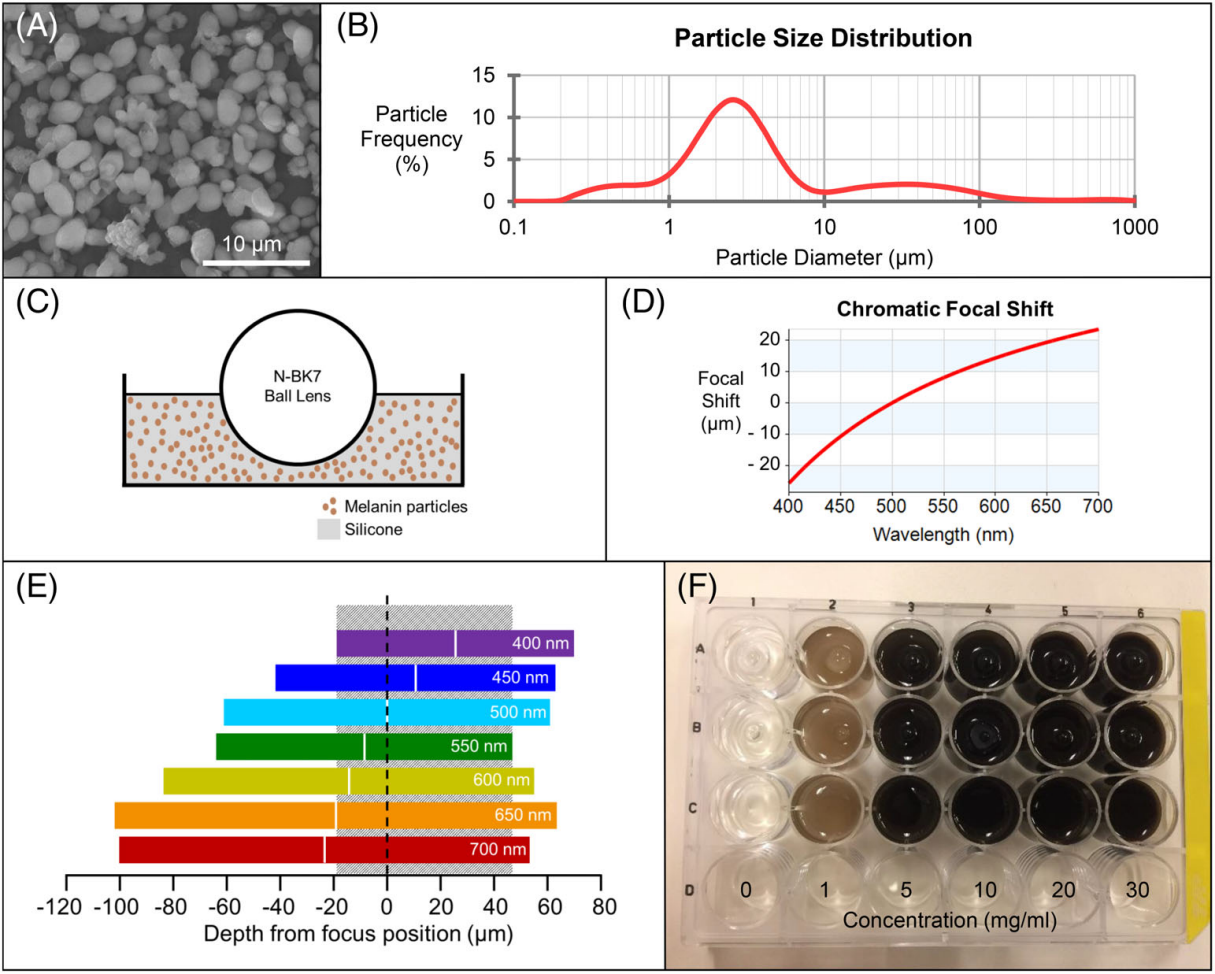
Ball lens rodent eye phantoms. A, Scanning electron microscope image of synthesized melanin powder. Note the small ellipsoid shape, similar to that found in the eye. B, Eumelanin particle size distribution as measured by laser diffraction analysis. C, Sketch of phantom (not to scale). D, Simulated chromatic focal shift as a function of wavelength for the 3 mm ball lens. E, Confocal parameter (length of bars) and focal points (white lines) for each wavelength for the 3 mm ball lens. The shaded area indicates the depth range over which the confocal parameter overlaps for all wavelengths. The zero position (dashed line) indicates the position of tightest focus for the reference wavelength of 500 nm. F, Photograph of the melanin phantoms. Top row: 6 mm ball lenses. Middle row: 3 mm ball lenses. Bottom row: no ball lenses. Concentration of melanin in silicone is indicated in the image for each column

Small ellipsoidal melanin particles, similar to those found in the eye 45, were fabricated from raw eumelanin (M8631; Sigma Aldrich). The eumelanin was dissolved in a 0.1 M sodium carbonate solution (37°C, 24 hours) before being re‐precipitated following addition of calcium chloride 45, 46. The solid particles were retrieved by centrifugation, washed using deionized water and freeze‐dried. The morphology of the resultant particles was evaluated by scanning electron microscopy (FEI Quanta 200), as demonstrated in Figure 2A. Using an aqueous particle suspension, the size distribution of the melanin particles was determined by laser diffraction analysis (Malvern Mastersizer 2000 with Hydro S) as seen in Figure 2B, revealing a peak particle diameter of 2.4 μm.

To create the ball lens rodent eye phantoms as sketched in Figure 2C, melanin particles were first mixed with a small volume of silicone (SYLGARD 184; Sigma‐Aldrich) and ground with a mortar and pestle to remove large agglomerates. This mixture, along with additional silicone to reach the desired volume, was then mixed for 2 minutes at 2000 rev/minute using a planetary mixer (ARE‐250; Thinky Corporation). Following this, hardener was added at a 1:10 hardener:silicone ratio and the mixing process was repeated. To remove air bubbles, an additional mixing step (1500 rev/minute for 4 minutes) was performed. The mixture was then divided between three wells, and N‐BK7 ball lenses of 3 and 6 mm diameter (43‐711 and 32‐746; Edmund Optics) were suspended in two of these wells before being left to dry for 24 hours. This process was performed six times for melanin concentrations ranging from 0 to 30 mg/mL, up to double the melanin concentration which was recently reported in RPE tissue 13.

To investigate the suitability of the N‐BK7 ball lenses for focusing, the propagation of visible light through the ball lenses was modeled using CODE V (2017, Synopsis, Inc.). As the system is spherically symmetric, only the longitudinal chromatic focal shift was investigated. By choosing the reference wavelength to be 500 nm, the shift in focus varies by ±24 μm across the whole wavelength range as shown in Figure 2D. The simulations also showed that the spot size for each wavelength is diffraction limited, and therefore the Airy radius was used for the following calculations.

The confocal parameter, *b*, was calculated for each wavelength from 400 to 700 nm:

(6) $$b=\frac{2\pi {\left(w_{0}\right)}^{2}}{\lambda },$$

where *w*_0_ is the beam waist, or half of the 1/*e* ^2^ diameter of the beam at its tightest focus. Figure 2E shows a plot of the confocal parameter at each wavelength (in 50 nm intervals), indicated by the length of the colored bars, while the focal points are indicated by white lines. As highlighted in the shaded gray area, there is a region of approx. 65 μm where all wavelengths can be considered in focus, that is, their confocal parameters overlap. The focus was therefore set in this area during the experiments.

The data shown in Figure 2D,E represents the 3 mm ball lens. For the 6 mm lens, the shift in focus is greater (±45 μm), however as the confocal parameter at each wavelength is longer, the region of focus overlap in depth increases. A photograph of the final phantoms can be found in Figure 2F.

### 3.2 Hyperspectral OCT

**Figure 3:**
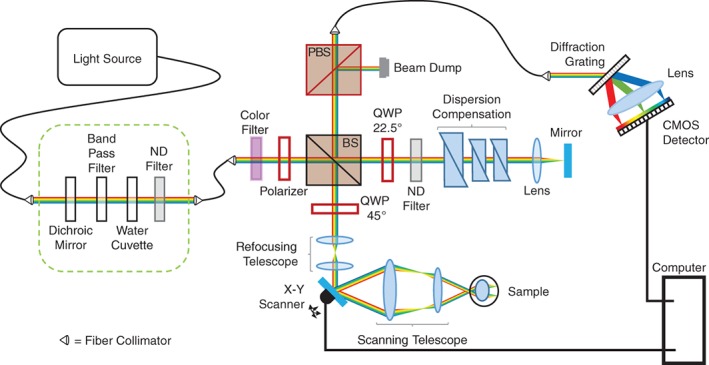
Diagram of the white light optical coherence tomography (OCT) system. BS, beam splitter; ND, neutral density; PBS, polarizing beam splitter; QWP Quarter‐wave plate

**Figure 4:**
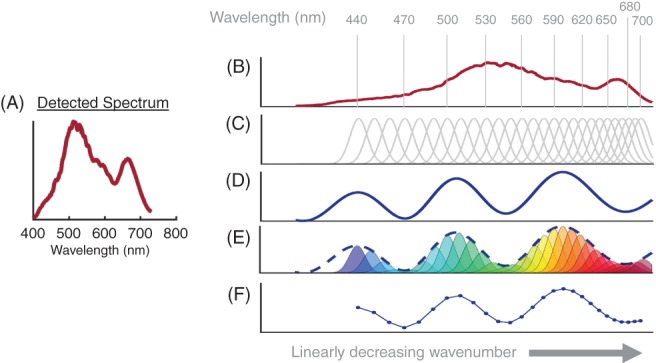
Detection of Mie backscattering coefficient by hyperspectral optical coherence tomography (OCT). (A‐C) are performed on every acquired A‐scan, while (D‐F) show a representation of backscattering coefficient detection. A, Reference spectrum as detected by the OCT spectrometer. B, Spectrum as in (A), resampled to be linear in wavenumber. C, Twenty‐seven Gaussian windows to create hyperspectral images in post‐processing. Central wavelength of windows increases linearly with wavelength (steps of 10 nm; full width at half maximum is constant with wavenumber to ensure a constant axial resolution). D, Example Mie backscattering coefficient, resampled to wavenumber (see 0.7 μm particle size in Figure 1A). E, Multiplication of Gaussian windows by the Mie backscattering coefficient. F, Integrating the area under each window results in an intensity dependence upon wavelength, whereby the characteristic intensity modulation can still be observed

Ball lens phantoms and the retinas of mice and rats were imaged using a modified version of the white light OCT system described in detail elsewhere 47. A diagram of the system, as it was used for this study, can be found in Figure 3. A magenta color filter was added to the system to suppress the green portion of the spectrum, allowing a higher relative intensity at the edges of the spectrum (Figure 4A). The total incident power on the cornea was 0.9 mW. The system operated at an A‐scan rate of 25 kHz, acquiring three‐dimensional images (512 × 400 A‐scans) with an en‐face area up to 1 mm^2^. To enhance the SNR in the rodent eye images, an additional two‐dimensional scanning protocol of 30 repeated B‐scans was introduced to allow averaging, and these data were used for the in vivo melanin visualization. The refocusing telescope was manually adjusted before each acquisition to ensure the tightest focus.

The acquired spectra firstly underwent background removal and reference intensity normalization before being remapped to be linear in wavenumber as shown in Figure 4B. The remapped data were then sub‐sampled into 27 wavelength bands (central wavelength = 440 to 700 nm, steps of 10 nm, bandwidth constant in wavenumber to ensure a constant axial resolution in tissue [6 microns], see Figure 4C). Second‐ and third‐order dispersion was compensated numerically for each wavelength independently 48 and each sub‐band was subsequently Fourier transformed. This resulted in a hyperspectral OCT stack of 27 images per B‐scan position, which were then manually axially registered (correcting for first‐order dispersion). For illustration purposes, Figure 4D‐F shows a representation of how the Mie backscattering coefficient is detected. As an example, the wavelength‐dependent backscattering coefficient taken from Figure 1B for the 0.7 μm melanin particle diameter in the rodent RPE is displayed in Figure 4D. By multiplication of Gaussian windows at the selected wavelengths by the Mie backscattering coefficient (Figure 4E), it is expected that the modulations introduced by the melanin particles can be observed using hyperspectral OCT (Figure 4F).

### 3.3 Animals

Both eyes of male non‐pigmented Oncins France Strain A (OFA) rats (N = 3, age = 13 weeks), pigmented Brown Norway (BN) rats (N = 3, age = 17 weeks) and mice from a C57BL/6 background (N = 3, age = 66 weeks) were imaged in this study. The animals were cared for by the staff at the animal facility of the Center for Biomedical Research, Medical University of Vienna. A 12‐hour photoperiod was observed, with food and water ad libitum. For the experiment, the animals were anesthetized using an isoflurane/oxygen mixture (4% isoflurane for the first 4 minutes to induce anesthesia and 2% thereafter, flow rate of 2 L/minute) and pupils were dilated using topically administered tropicamide. The eyes were kept moist during the experiments using artificial tear drops. During measurement, the animals were placed in a homemade translational/rotational stage that enabled alignment of the eye with respect to the measurement beam. The duration of anesthesia was less than 45 minutes in total for each animal resulting in a fast wake‐up process, with full mobility regained within approximately 1 minute. All experiments were performed in accordance with the ARVO Statement for the Use of Animals in Ophthalmic and Vision Research and Directive 2010/63/EU. Ethics protocols were approved by the ethics committee of the Medical University of Vienna and the Austrian Federal Ministry of Education, Science and Research [BMBWF‐66.009/0216‐V/3b/2018 (mice); BMBWF‐66.009/0183‐V/3b/2018 (rats)].

### 3.4 Image analysis

For hyperspectral image analysis, the maximum intensity projection (MIP) was first taken across all 27 wavelength images. A watershed transformation 49 was then performed on the MIP image to define catchment basins on the order of single speckles. To avoid oversegmentation, a minimum basin area of 8 μm^2^ (corresponding to just below the image resolution) was defined. The catchment basins were used to segment the original 27 wavelength images and the mean intensity values within each basin were calculated for each wavelength. These steps were performed to remove the effect of the wavelength‐dependent speckle patterns on the hyperspectral analysis.

**Figure 5:**
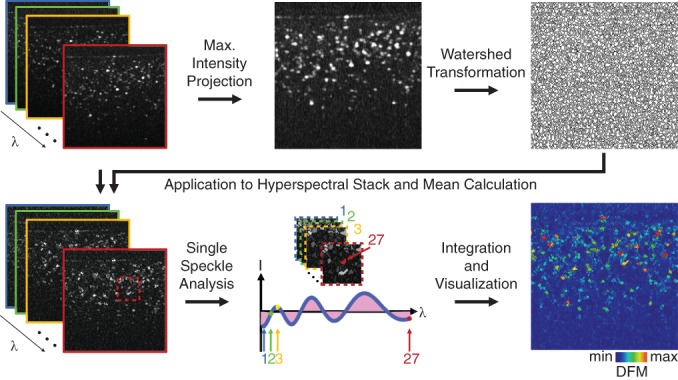
Illustration of hyperspectral image analysis pipeline on one B‐scan. A maximum intensity projection is performed across all wavelengths and then the watershed transformation is applied to bin the pixels into speckles. The resulting delineations are then applied to the original hyperspectral stack, and a mean intensity value for all pixels within the binned speckle is calculated for each wavelength. For each speckle, the mean intensity across all 27 wavelengths is calculated and subtracted from each wavelength channel. The resultant spectrum is plotted as a function of wavelength. For melanin visualization, the area under the curve is calculated by numerical integration and the values are assigned to a color scale and the speckle location is remapped to the original image, resulting in a “deviations from mean” (DFM) map

To test for Mie behavior, the mean intensity value across all 27 wavelengths was calculated and then subtracted from each wavelength individually. If the backscattering of the sample was largely independent of the incident wavelength, that is, in the optically scattering region where the particle diameter is much larger than the wavelength, the intensity values should cluster around this zero line. However in the case of Mie backscattering, the intensity of the catchment basin varies as a function of wavelength, resulting in larger deviations from the mean (DFM) values. As a method of visualization, the numerical integral of the absolute value of the deviation intensity, *I*_dev_, with respect to wavelength, *λ*, was calculated,

(7) $$\mathrm{DFM}=\int_{\lambda_{1}}^{\lambda_{27}}\left|I_{\mathrm{dev}}\left(\lambda \right)\right|\mathrm{d\lambda},$$

with larger numbers corresponding to greater deviations. These numbers were mapped back to the watershed catchment basins from which they originated, in a DFM image. All calculations were performed on images with a linear intensity scale. A diagram of the image analysis pipeline can be seen in Figure 5.

### 3.5 Electron microscopy

For ultrastructural analysis, eyes from pigmented mice (C57BL/6J; Jackson Laboratory, Bar Harbor, Maine) and rats (Brown Norway; Charles River Laboratories, Wilmington, Massechusetts) were fixed in 2.5% glutaraldehyde, 4% paraformaldehyde in 0.1 M phosphate buffer and embedded in Epon. Series of ultrathin sections (70‐90 nm) were collected on copper grids, contrasted with lead citrate and uranyl acetate and viewed with an electron microscope (EM900; Zeiss). Images were acquired using a 1024 × 1024 pixels frame transfer CCD camera (Tröndle TRS, Moorenweis, Germany) and Image SP Software (SYS‐PROG, Minsk, Republic of Belarus), stitched using the Distortion Correction Plugin 50 in ImageJ and adjusted for brightness and contrast with Adobe Photoshop CS6.

## 4 RESULTS

### 4.1 Phantom measurements

**Figure 6:**
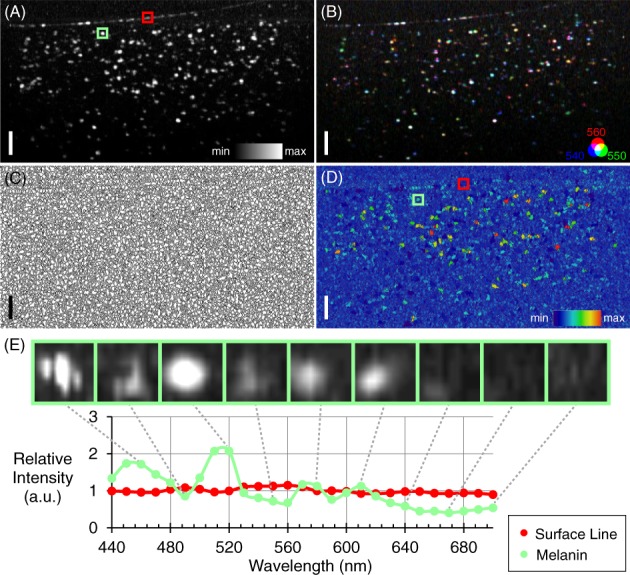
Melanin particle detection by hyperspectral optical coherence tomography (OCT). Example shown here is 10 mg/mL melanin in silicone with the 6 mm ball lens. A, Maximum intensity projection across all wavelengths. B, RGB image encoding of three wavelength channels: **Blue** 540; **Green** 550 and **Red** 560 nm. The presence of the range of colors indicates that the coefficient of backscattering is different for the different wavelengths. C, Watershed boundaries of the image. D, Deviations from mean image. The surface line is much less visible in this representation, indicating that the coefficient of backscattering is less wavelength‐dependent. E, Examples of relative intensity as a function of wavelength within single watershed boundaries for both the surface line (from the region indicated with the red box in [A] and [D]) and the melanin (from the region indicated with the green box in [A] and [D]). Small insets show the reflectivity profile of the granule at selected wavelengths. Scale bar = 50 μm

All melanin phantoms were imaged by the white light OCT system, and the hyperspectral analysis was performed on these images. A MIP across all 27 wavelengths can be seen in Figure 6A (10 mg/mL, 6 mm ball lens). It was immediately clear that the MIP contained many more particles than any individual wavelength. To demonstrate this visually, an RGB image was created (Figure 6B) consisting of three consecutive wavelength channels encoded into the three colors: 540 nm in blue, 550 nm in green and 560 nm in red. The lens‐silicone surface appears more white than the melanin particles. This suggests that the backscattering of this surface is less wavelength‐dependent than the particles, which would be consistent with Mie theory.

For further analysis, the watershed transformation was applied (Figure 6C) and the DFM image, shown in Figure 6D, was calculated. This again shows the low variation in backscattering coefficient at the lens‐silicone interface, while highlighting the differences in the melanin granules. The difference in the relative OCT signal intensity as a function of wavelength between the surface line and the melanin granules can be seen in Figure 6E. Also displayed is one of the melanin granules at a selection of the individual wavelengths. Not only does the intensity change, but the apparent location and shape of the granule shifts too. This is due to the scattering profile of the individual granules, demonstrating the need for the watershed method.

**Figure 7:**
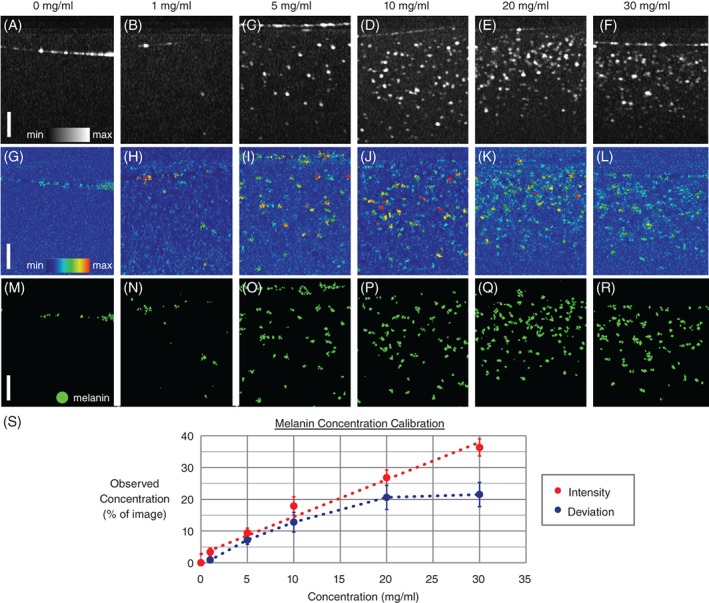
A‐F, Maximum optical coherence tomography (OCT) intensity projections across all wavelengths. (G‐L) Deviation from mean (DFM) B‐scans of melanin ball lens phantoms as acquired by hyperspectral OCT. The percentage (by area) of the image which contained pixels above 3× the standard deviation of the noise was calculated in both intensity and DFM. A binary representation of pixels above the DFM threshold can be seen in (M‐R). S, Melanin concentration calibration graph. Error bars indicate standard deviation across 400 B‐scans. While the intensity information shows a linear increase in concentration across the measured range, the DFM plot levels off at higher concentrations. Scale bars = 50 μm and apply in (A‐R)

After proving that it was possible to visualize the melanin granules, the possibility of determining the absolute melanin concentration using this method was investigated. Figure 7A‐F shows MIP B‐scans across all wavelengths for known concentration values ranging from 0 to 30 mg/mL. The corresponding DFM plots can be seen in Figure 7G‐L. For all concentrations, the same trend as in Figure 6 was observed whereby the surface line is less visible in the DFM images. For both datasets, the surface line was excluded and the percentage of pixels above 3× the standard deviation of the noise was calculated over 50 μm in depth (smaller than the confocal parameter of the mouse eye phantom). Figure 7M‐R shows the thresholded DFM images, with pixels above the threshold in green. Figure 7S shows a map of the observed percentage of high intensity pixels based on both the MIP intensity data and the DFM plots. The data plotted is the mean and standard deviation over 400 B‐scans. When considering the intensity alone, the observed concentration linearly increases as a function of actual concentration across the investigated range. However this is not the case for the DFM measurements. The observed concentration follows a linear trend (with a small offset) until around 10 mg/mL, but then starts to reach a plateau. This effect can also be seen qualitatively from the images.

### 4.2 In‐vivo RPE melanin visualization

**Figure 8:**
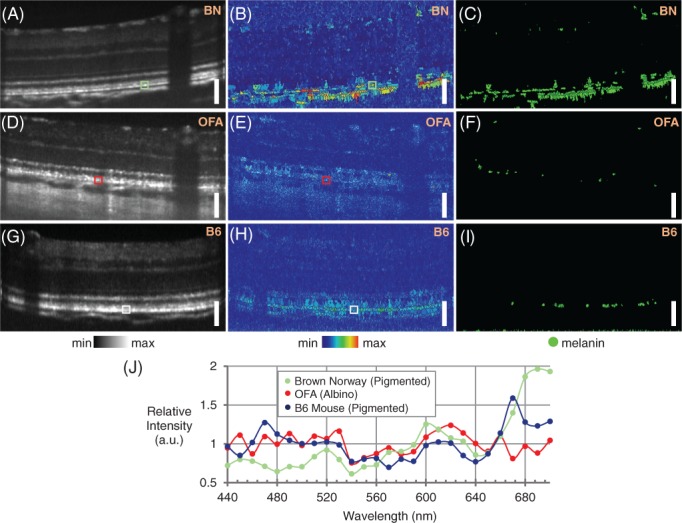
In vivo results of melanin visualization by hyperspectral optical coherence tomography (OCT). A, Intensity; B, Deviation from mean (DFM) and C, Thresholded DFM images in the Brown Norway (BN) rat retina. In the DFM plots, high signal is only observed in the retinal pigment epithelium (RPE) and the choroid, both of which are melanin‐containing. D, Intensity; E, DFM and F, Thresholded DFM in the albino OFA rat retina. The DFM image in (E) does not contain many pixels above the threshold (F). G, Intensity; H, DFM and I, Thresholded DFM in the retina of a mouse from a C57BL/6 background (B6). The signal in the RPE is much less pronounced than in the case of the Brown Norway rat. J, Examples of relative intensity as a function of wavelength from within single watershed boundaries for the pigmented Brown Norway rat (from the region indicated with the green box in [A] and [B]), the albino OFA rat (from the region indicated with the red box in [D] and [E]) and the the pigmented mouse (from the region indicated with the white box in [G] and [H]). Scale bars = 100 μm

To test if the same effect could be observed in vivo, eyes of three pigmented BN rats (example can be found in Figure 8A‐C), three non‐pigmented OFA rats (example in Figure 8D‐F) and three pigmented mice from a C57BL/6 background (example in Figure 8G‐I) were also imaged with the white light OCT system. Figure 8A shows an example of a MIP across all wavelengths for the BN rat, while Figure 8B shows its corresponding DFM image. In the DFM image, the melanin‐containing RPE is highlighted as a layer which shows deviation unlike the rest of the retina. Some regions of the choroid also appear to show increased DFM. These effects are also highlighted in the thresholded binary DFM image in Figure 8C.

The six OFA albino rat retinas were imaged to test if this effect was specific to melanin (example intensity B‐scan shown in Figure 8D, DFM image in Figure 8E) and thresholded DFM in Figure 8F). Displayed on the same colorscale, the DFM image did not highlight the RPE in any of the images, which can be observed by the thresholded DFM image (Figure 8F). In Figure 8J, examples of the intensity as a function of wavelength for single watershed‐created pixels can be seen. For the non‐pigmented case (indicated by the red box in Figure 8D,E), the deviations are small and fluctuate around the mean intensity. However for the BN rat (indicated by the green box in Figure 8A,B) the deviations are much further from the mean intensity values.

**Figure 9:**
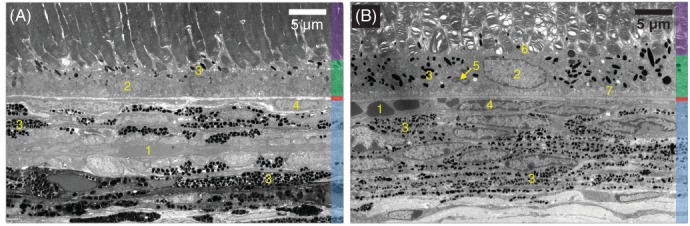
Transmission electron microscopy images of the Brown Norway rat retina (A) and the C57BL/6 mouse (B). Colors indicate layers: **Purple** Rod outer segments; **Green** Retinal pigment epithelium (RPE); **Red** Bruch's membrane; **Blue** Choroid. Numbers indicate features: **1** Red blood cell. **2** RPE cell nucleus. **3** Melanin granules. **4** Endothelial cell. **5** Mitochondria. **6** Apical microvilli. **7** Basal microvilli

Imaging was also performed on three pigmented mice from a C57BL/6 background (six eyes imaged, one eye excluded due to motion artifacts) to see if the melanin in the RPE could also be observed here (examples in Figure 8G‐I). However in images of all five mouse retinas, the DFM signal in the RPE was very similar to that of the albino rat (example shown in Figure 8J with a pixel indicated by the white box in Figure 8G,H), indicating that it was not possible to detect the melanin with this technique. To investigate this further, ultrastructural analysis of both the BN rat retina and the C57BL/6 mouse was performed. From the transmission electron microscopy images in Figure 9, it can be seen that the concentration of melanin granules in the mouse RPE is much greater than in the BN rat. When considering the laser spot size on the retina during OCT imaging (the lateral resolution is estimated to be approx. 2–3 μm), the beam would likely only hit one or two melanin granules per A‐scan in the BN rat retina. This would not be the case in the mouse, as the laser beam would hit several granules of different sizes, each of which contributes to the observed modulations.

## 5 DISCUSSION

Eumelanin is highly absorbing and therefore provides a strong contrast in photoacoustic tomography and PT OCT. Previous simulations have therefore focused mainly on the absorption characteristics, treating the whole RPE as one homogeneous layer 51. However, this only applies in the limit of a high melanin concentration. Since eumelanin is a heterogeneous macromolecule comprising numerous cross‐linked polymers (5,6‐dihydroxyindole [DHI] and its 2‐carboxylated form 5,6‐dihydroxyindole‐2‐carboxylic acid) 52, it forms pigmented granules in the RPE which vary in melanin content. For low melanin concentrations and small probing beam diameters, the overall size and shape of the pigment is the dominating factor for changes in the backscattering properties. It is therefore important to consider methods for melanin visualization, also when the concentration is low.

Hyperspectral OCT has successfully demonstrated its ability to detect varying backscattering coefficients as a function of wavelength both in melanin phantoms and in vivo. The phantoms used in this experiment contained melanin particles which were similar in shape to those found in the rodent RPE, albeit a little larger on average. Nevertheless, they fell into the detection limits of the hyperspectral OCT system. To mimic the rodent eye, a ball lens was used to provide the focus. For the OCT system used in this study, the fact that the focus always falls behind the back surface of the ball lens was not a problem as the system contained an additional manually adaptable refocusing telescope. However for a system without this capability, a more sophisticated lens configuration would be required to achieve a tight focus.

A linear relationship between the pixels above the threshold in the DFM plots and the actual concentration of the sample indicates that with calibration, the concentration of melanin granules in the phantoms could be measured. In the phantom measurements, the concentration could also be calculated directly from the conventional OCT images, as the number of hyperscattering pixels also scales linearly with the concentration. At the lens‐silicone interface, the DFM values are generally low indicating that the backscattering coefficient is largely independent of the incident wavelength. High DFM values at the interface are mostly attributed to saturation of the signal in the intensity images, although there will also be some melanin granules exactly at the boundary too. However, as the dominant cause of the high DFM values is the saturation, the interface line was excluded from all concentration measurements. In the in vivo case, the melanin is not the only scatterer present, demonstrating the need for the DFM method. The enhanced contrast given in the DFM images is assumed to be specific to the melanin, as there is no increased signal in the albino rat RPE. However the phantom measurements show that the DFM method reaches a saturation point—the values become generally lower at high concentrations. This indicates that the DFM images, and therefore also the ability to detect Mie scattering by hyperspectral OCT, is only applicable at low concentrations with the current apparatus. Using this method, it could also be possible to estimate the size of the particles by correlating the spectral shape back to the Mie theory simulations. It would, however, be challenging to correlate this directly as the size and shape varies from one particle to another, and it cannot be ruled out that the observed signal does not come from only one melanin granule. This could cause some aliasing artifacts. However for qualitative visualization purposes, as the modulations are still observed in the spectrum, the presence of melanin can be identified without knowing the exact dimensions of each individual granule. In order to make quantitative statements regarding the size and shape of the particles, a more sophisticated particle backscattering model would be required which includes all possible sizes and shapes of melanin granules in the RPE, for example by the MONTCARL software 53, 54.

In the in vivo rodent retinas, differences between albino and pigmented rodent RPE were identified, but only in the animals where the melanin concentration is low as is the case in the BN rat. Owing to motion artifacts, image averaging becomes more challenging and therefore the modulation signal is generally less pronounced when compared to the phantoms, but the effect can still be observed. The example spectra shown in Figure 8J show a higher intensity of modulation for the BN rat than for the non‐pigmented rat or the mouse. The spectrum in this example shows an increased intensity after 640 nm, perhaps corresponding to a melanin granule similar in size/shape to the 1 μm spherical granule shown in Figure 1B. Each individual granule, however, displays a different spectral signature, corresponding to the different sizes and shapes of the granules.

As the entire spectral information is acquired simultaneously in spectral domain OCT, only a single measurement is required to obtain each hyperspectral depth profile. No further spectral image registration is required other than that caused by dispersion. This results in a hyperspectral imaging stack of all retinal layers—not only the RPE. Consequently, the spectral data could be used for detection of other chromophores, such as oxy‐ and deoxy‐hemoglobin in the blood vessels. Red blood cells themselves are too large to produce a pronounced Mie effect in this wavelength range and the absorption of single pixels within the vessels is not strong enough to increase the DFM above the threshold. However since the spectral information is already present, a more traditional spectroscopic analysis could be applied to the data for this purpose 29, 30, 31.

There is also some DFM signal from the choroid in the BN rat, but not in the other rodents. This is most likely due to a combination of the increased density of the melanin in the choroid as compared to the RPE (see Figure 9), the change in size of melanin particles between the regions (in the mouse) and the increase in light penetration to the choroid in the BN rat due to the lower RPE melanin concentration. In order to improve the resolution of the images to see this effect in more pigmented RPEs (such as in the mouse), or in the choroid, higher axial, lateral and spectral resolution would be required. A higher spectral resolution would increase the range of particle diameters which could be observed, which may also be useful in other pigment‐containing tissues. However there is an inherent trade‐off between axial and spectral resolution in OCT. With the broad bandwidth used in this study, filtration by 27 Gaussian windows was sufficient to observe the modulations while maintaining a high enough axial resolution (6 μm) to resolve the RPE. Methods to overcome this limitation have also been proposed 55, 56, 57 and could also be applied in similar studies. Since the lateral resolution is dependent upon the numerical aperture of the imaging lens, i.e. the rodent eye itself, it will differ slightly from one animal to another. In this system, it is estimated to be 2–3 μm, however this will also vary as a function of wavelength and therefore contribute to changes in the speckle pattern throughout the hyperstack. Integrating adaptive optics into the system could help to solve this 58, although this would add further complexity to the system.

More generally, this study raises further questions for OCT imaging. If the acquired OCT spectra consist of irregular sample intensity across the wavelength range, this would reduce the axial resolution and perhaps cause side lobes to be present in the images. The speckle shape is also different for each wavelength, and the point spread function is modulated in depth 35, making traditional hyperspectral image analysis techniques more challenging in hyperspectral OCT. Pixel‐based approaches are not suitable as the intensity data per single pixel does not give an accurate representation of the total backscattered intensity. For this study, the watershed segmentation method was utilized to overcome this limitation. Although a well‐established technique in image segmentation 59, the application of the watershed transformation to OCT data is relatively new 60, 61. This method is, however, sensitive to speckle. The speckle patterns were suppressed in this study by using the MIPs over the 27 independent wavelengths. Application of the watershed transformation to standard OCT images would require another means of speckle reduction, for example the Bayesian non local means filter as used by Girish et al. 60.

Owing to the spectrometer configuration, the recorded data was not measured linearly in wavenumber. This resulted in a wavelength‐dependent sensitivity and roll‐off which also had to be corrected for. This could be improved by designing a spectrometer based on a grating‐prism setup 62, 63, 64. Choosing to sample the spectra to be linear in wavelength for the hyperspectral images also resulted in a lower system sensitivity to higher frequency Mie modulations at shorter wavelengths, as the wavenumber sampling density increases with increasing wavelength. The non‐linear wavenumber sampling is, however, also an advantage. In the case of aliasing, some modulation is still very likely to be observed and while this would not be suitable for particle size estimation, qualitative identification of the particles is still possible. While the image analysis method was designed to remove signal contributions due to laser source noise and spectrometer noise, the DFM method is sensitive to the wavelength‐dependent sensitivity roll‐off which occurs due to the unequal wavenumber sampling. To minimize such effects in vivo, the images are acquired using enhanced depth imaging 65, that is, the RPE is closest to the zero delay, with the inner retina further away.

When considering RPE melanin concentration alone, the mouse may be a better model of the human than the BN rat as the concentration is higher 3. For visualization of melanin in these high concentrations, imaging methods based on melanin absorption and/or depolarization may be more appropriate than those based on backscattering. Nevertheless, there is not yet a consensus on the absorption, scattering and depolarizing properties of melanin in the RPE, and the average melanin concentration of the human RPE quoted in literature varies by up to three orders of magnitude 44, 51, 66. In order to develop a greater understanding of the complex relationship between the melanin in the RPE and visual function, a detailed optical analysis consisting of absorption, backscattering and polarization analysis is required, and this study is a step towards that goal.

## 6 CONCLUSIONS

It has been shown that with some prior knowledge of the granule size range and at low concentrations, it is possible to detect the presence of melanin using hyperspectral OCT. This was demonstrated both in phantoms and also in vivo in the BN rat retina. For the CL57BL/6 mouse, the melanin granules are too densely packed for the resolution of the system, but in that case it is likely that the absorption/depolarization properties dominate over the backscattering properties and therefore methods such as photoacoustic tomography may be more suitable for concentration measurements. Nevertheless, in order to fully understand the signals which come back from in vivo retinal imaging systems, a complete model consisting of absorption, scattering and polarization properties is required.

## CONFLICT OF INTEREST

The authors have no potential conflict of interest to disclose.
